# Supplementary material for: Factors driving underweight, wasting, and stunting among urban school aged children: Evidence from Merawi town, Northwest Ethiopia
Source: PLOS Glob Public Health. 2023 Jan 24;3(1):e0000586. doi: 10.1371/journal.pgph.0000586 (PMC10021509; doi:10.1371/journal.pgph.0000586)
Supplement: Attachment — Submitted filename: Response to Reviewers.docx [file pgph.0000586.s001.docx]

| **Response to reviewers’ comments** | |  |
| --- | --- | --- |
| **Title**: Factors driving underweight, wasting, and stunting among urban school aged children: Evidence from Ethiopia | |  |
| **Reviewer #1:** **Feedback to authors** | **Authors’ response** | **Authors’ comments** |
| Thank you very much for partaking in this essential work. This cross-sectional study probed factors driving underweight, wasting, and stunting among urban school-aged children: Evidence from Ethiopia. The issue is still requiring attention and the findings are remarkable. However, I have a few concerns that could be clarified and amended for better understanding. | Thanks for your valuable comments, We have addressed your specific concerns in the following sections. |  |
| Comments and questions:  • On the abstract part: 1. Background - line 7 and result- line 3, the word “determinants”, is better replaced by another suitable action verb, since you have conducted a cross-sectional study. Similarly, on the same line, the phrase “…identify and assess…” should be rearranged. | As suggested, we have now rearranged words in the abstract (***page 2-line number 8 and 16 in green colour)*** |  |
| 2 The method of sampling should be specified as well. | As suggested we have now specified the method of sampling (***page 2-line number 12, coloured in green).*** |  |
| • On the background part: ϖ The first paragraph reference number 1 and 4 talk about under-five children instead of your focus area which is school-age children. | Thanks for your comments. Please see our response and comments in the next column. | We have showed the level of malnutrition in children globally first and then discuss school age children in the process. We have now cited references related to school age children. |
| ϖ In the second paragraph, there is a mix of ideas i.e. initially you stated global magnitude of malnutrition (first line), then about SAM (second line), consequently about risk factors (from line 3 to 5), finally global magnitude of malnutrition again (from line 6 to 8). Therefore, it is better to develop paragraphs with one topic sentence and other supporting sentences separately. | Following your comments , we have now updated this paragraph and made it clearer for readers (see ***page 3, coloured in green).*** |  |
| ϖ The 3rd paragraph – reference number 8 does not match on the list of references (EDHS 2011 instead of EDHS 2016). And these children are under-five as well. | We have updated the information in the 3^rd^ paragraph (see changes in green colour) |  |
| ϖ The 3rd paragraph – lines number 4 and 5 (… 700,000 pregnant and breastfeeding women …) is out of the scope of this study. | We have now deleted lines 4 & 5. |  |
| ϖ The 3rd paragraph – line number 6 (… the rate of this reduction in under-five …) better to focus on school-aged children. | We have updated the information as suggested (see coloured in green) |  |
| • Methods and materials ϖ Variables of the study − You said your dependent variable is the nutritional status (underweight, wasting, and stunting) among under-five children. Why? | We have now corrected and provided necessary explanation as to why?’ (***page 5-line number 23 in green colour)*** |  |
| − On independent variables line number 6 (environmental factors such as Unhygienic …) requires edition. Additionally, what do you mean by “birth size” on line number 7? And what is its relevance, since it is not included in your result part? | Thanks for these comments. we have now updated the word to ‘Unhygienic living condition’ to ‘sanitation’ ***(page 6-line number 1-2, cloured in green)*** |  |
| − On page 6 the 1st paragraph line number 4 – you stated that “the variable (nutritional status) is measured by the level of underweight, wasting and stunting, diet habits and household food security.” How did you measure and operationalize household food security)? And it is not included in your result part. Thus, it is better to remove diet habits and household food security. | As suggested we have now removed ‘ diet habits and household food security’. |  |
| − On page 6 the 2nd paragraph, it is already described on page 5 under independent variables. Therefore, what is the value of repeating it? | Thank you, we have now deleted this paragraph as it is redundant. |  |
| ϖ Data analysis, result, and discussion − First paragraph lines number 3 and 4 – do you mean AORs are used for presenting multivariable logistic regression analysis output? | Yes, we do. have now updated this information in the revised manuscript ***(page 7-line number 14-16, coured in green).*** |  |
| ϖ Socio-demographic characteristics − On page 7, 392 response rate is not consistent with the one indicated throughout the tables (396). | It now corrected and made this information consistent. The response rate is 93.6% ***(page 7-line number 19 coloured in green).*** |  |
| ϖ Factors driving malnutrition − Before identifying factors driving malnutrition, you have to put the nutritional status of school-age children in terms of underweight, stunting, and wasting. | We have now presented the nutritional status of children in the first paragraph of the seam subheadings ***(page 8-line number 17-20, coloured in green).*** |  |
| − Paragraph one line one – you said that “… bivariate analysis…” , do you mean bivariable logistic regression analyses? | Yes, we have now corrected it accordingly (***page 8-line number 19-20, coloured in green).*** |  |
| − The dependent variable underweight (weight for age) is the composite measure of malnutrition. But, the age of children is considered a predictor of being underweight, which is inappropriate. Therefore, the analysis has to be done again. | Please see our comments in the next column. | We appreciate your view that age is included in the determination of underweight; however, this age a continuous variable and specific for each child and not adjusted for all children. Please note an increase in age does not always mean decrease in weight of a child as it has many cofounding factors associated with it. We, thus analysed the age as a predictor variable after we categorized it in two groups. That means our intention is to show the age group that have prevalent underweight for better recommendations. |
| − Similarly, since stunting is measured using height for age, using age as an independent variable is inappropriate. Therefore, the analysis has to be done again. | Please see our comments in the next column. | Please note that here our intention is also to show the age group having higher rates of stunting. |
| − Table 3, 4, and 5 – all of the confidence intervals constructed are wide. This is related to using small sample size, hence, to come up with better analysis output, categories with very small values shall be revised and merged. | Thank for this comment. Please see our response and comments in the next column | We analysed the results with different alternative categories of the independent variables and we believe this is the appropriate model best explaining our assumptions. We understand that some confidence intervals are wide, this is because there is sometimes a sudden increase in height school age children that could affect all parameters like weight. |
| • Discussion ϖ Paragraph one, lines 4, 5, and 6 – “This could be attributed to the fact that in the study area, child eating habits were extended to almost three times per day, …”. Have you come across other areas children’s feeding habits to say this? “… while fasting occurred in the early age group, and there was an absence of school-based feeding.” Do you think these ideas support your argument for having lower underweight compared with the aforementioned areas? | Thanks very much for your nice view here, we have now updated it to support our findings (***page 10-line number 17-19, coloured in green).*** |  |
| ϖ Third paragraph, line 2 – What about the timing of the research they conducted? For example, reference numbers 16 (2008) and 19(1998). It is better to use recent references for a reasonable comparison. This comment is also applied to the prevalence of stunting since similar references are used (Paragraph three, line 10). | Thanks for this we have now compared the results with the recent findings. |  |
| • Concluding remarks ϖ Line one – “… arena …”, do you mean area? | Thanks, we intended to mean area and we have now corrected it on ***page 5-line number 13 (see coloured in green)*** |  |
| **Reviewer #2: Feedback to authors** | **Responses** |  |
| The grammatical and sentence structure needs modification as general comments. | Thank you very much for your time and expertise for reviewing our manuscript and your valuable comments for improving our work. |  |
| 1. Abstract section ¬ Results: ………….. key determinants of underweight, and the gender of the child was significantly associated with wasting [AOR= 0.455 (0.224, 0.927)]. The word determinants are better replaced by associated factors since your study design was cross-sectional. ¬ Conclusion: make clear the possible early interventions that you intend | Thank you for this comment, we have now rearranged words in the current version as indicated in the abstract (***page 2-line number 8 and 16, coloured in green).*** |  |
| 2. Background ⎫ Take updated data about the prevalence of nutritional status with updated references worldwide ⎫ Take the Ethiopian mini-EDHS 2019 data ⎫ Explain the interventions regarding the Ethiopian context of school health and nutrition initiatives | Thank for your comments. As suggested we have now updated the introduction based on your comments. |  |
| 3. Methods and materials ⎫ During your data quality assurance of questionnaire, you mentioned that as you pre-tested in 5% of the calculated sample size what was your chrome batch alpha? ⎫ During your data analysis specify your cut of the p-value for eligibility to be screened for the multivariate logistic regression model | Thanks for this comment. The chronbach’s alpha coefficient was 0.76 and the cut of values of p-values and the ethics approval number are now included in the revised version. |  |
| 4. Result ¬ Maternal and child health-related characteristics variables in Table 2 need operational definitions of how they were measured: ϖ A total of 360 (90.9%) mothers had access to child nutrition education, ϖ (255 (64.4 %) took vitamin A supplement up) ϖ 55 (13.9%) children engaged in work, ϖ 61 (15.4%) experienced water shortage for cooking, ϖ 367 (92.7%) used pipe water, ϖ 372 (93.9%) regularly kept child hygiene ϖ 323 (81.6%) regularly washed their hands, ϖ 352 (88.9%) cut their nails, ϖ 323 (81.6%) used iodized salt, ϖ 288 (72.7%) had a modern latrine facility | Thank for your comment, we have now defined these variables below the table with key notes. |  |
| NB : ¬ Accessibility of child nutrition education how it was measured? ¬ vitamin A supplementation: from the standard of supplementation how many times has the child has supplemented from his/her age ¬ modern latrine facility: what were the criteria used to determine a latrine to be said modern? The same is true for the above-mentioned factors | Thank for your comment. we have defined these variables below the table with key notes. |  |
| 5. Discussion ¬ Give evidence-based expiation for gender and size variables | Thank you, evidences are now cited that support our explanation in the discussion section (***see coloured in green, Page 9 – line numbers 17 - 19***). |  |
| 6. Conclusion ¬ Indicate specifically the possible interventions for concerned body based on your findings. | Thank you for this, we have now tried to make our conclusion more specific in line with our research findings(***see coloured in green, Page 10 – line numbers 24 – 30 and page 11 – Line number 1***). |  |
