# Supplementary material for: Factors driving underweight, wasting, and stunting among urban school aged children: Evidence from Merawi town, Northwest Ethiopia
Source: PLOS Glob Public Health. 2023 Jan 24;3(1):e0000586. doi: 10.1371/journal.pgph.0000586 (PMC10021509; doi:10.1371/journal.pgph.0000586)
Supplement: Attachment — Submitted filename: Responses to reviewers.docx [file pgph.0000586.s002.docx]

**Factors driving underweight, wasting, and stunting among urban school aged children: Evidence from Merawi town, Northwest Ethiopia**

| **Reviewers 1** | **Response from Authors** |
| --- | --- |
| Kamal and colleagues examined factors associated with underweight, wasting and stunting among urban school aged children in Merawi town in Ethiopia based on primary data collected between the period 1^st^ April to 15^th^ June 2018. The study attempts to address a public health challenge, especially in developing countries like Ethiopia. Studies of this nature are relevant to policymakers and other stakeholders for informed decision making.  See more details below: | Dear reviewers thank you for your suggestions and time for reviewing our manuscript. We really appreciated it. |
| **Background**  The background provided by the authors is satisfactory. | Thank for this. |
| **Methods and materials**  The data collection procedures are satisfactory. | Thanks very much. |
| The authors applied binary logistic regression model to identify factors associated with the nutritional status of children. However, they failed to provide information on how they assessed multicollinearity of the independent variables in their multivariable binary logistic regression model. The authors could use generalised variance inflation factor to achieve this. Also, they did not assess the goodness-of-fit for their final multivariable binary logistic regression model interpreted in this study. The Hosmer-Lemeshow test could be used to achieve this. These are important for model diagnostic assessment to examine how well the model fits the data and to correct for any multicollinearity. | We really appreciated your comment on the analysis part. We examined for collinearity. The results are in acceptable range VIF less than five. Also, non-significant Hosmer-Lemeshow test was achieved. We have now described the results of these tests in the analysis part (page-7, line-197-200) |
| In adjusting for the associated factors, the authors could have considered the three (3) Kebele (small administrative units) as one of the potential predictors of nutritional status of these children. This is important because children from different Kebele might experience different nutritional outcomes, and this could inform targeted interventions for each of the Kebele in the study.  In the title, the authors mentioned urban school aged children but mentioned under-five children under the ‘dependent variable’ session. ‘Urban school aged children’ is not the same as ‘under-five children’. The authors should clarify this.  There are some typo errors that the authors should correct. For example, unhygienic at page 6. | We appreciate on your comment on including kebeles in the final model. However, the city has only three kebeles (small administrative units) only used for administrative purposes and the population among the kebeles are homogeneous, there is no difference among them.  For the typo errors and the consistency of ‘Urban school aged children’ we have now corrected it in the current version.  (Page-6, line-160; 166) |
| **Results**  The results presented are supported by the data, but the issues raised in the methods session should be addressed to improve the results.  Table 1 should be better presented as ‘Stunted’ versus ‘Not stunted’ against the socio-demographic variables in Table 1, and same be done for underweight and wasting. This is more informative than what is currently presented in Table 1. | We have now made the table to be more informative by taking your suggestions to includes descriptive for stunting, underweight and body mass index. Thanks for this (see table 1) |
| **Discussion**  The discussion appears good. However, the issues raised should be addressed to improve the quality of the paper. The manuscript will also benefit from some proofreading to improve the message. | We the authors and our colleague (who have experience scientific writing) have tried to make clear the write up. We believe it reads well now. |
| **Reviewers 2** |  |
| **General comment**: The knowledge gap is not clearly described. Example, very few literatures consulted, most of these literatures are related to children under the age of 5 years (not similar to population of current study), most of the literatures are out-dated, and relevant local studies are excluded/not reviewed. Moreover, the study is also underpowered. There are typographic and English grammar errors that need revision. What is new in this work? | First, we want to appreciate your detailed comment and suggestions to improve our manuscript. It helped us to improve our manuscript clearly.  Saying this, we have tried to make the writing clear to read, and corrected typo errors as well updated references.  We believe the result will at least provide some information about nutritional status and related factors among school age children in the study area, which could help local health workers and relevant bodies about the situations. Besides, it can serve as a comparison with other areas.  (Page-4, starting from line-91) |
| Comment on the details:  Topic:   - Looks as if the investigators are examining the problem under study using analytic study design such as case-control study. Furthermore, the paragraph ‘Evidence from Ethiopia’ is not appropriate because this piece of information is from small rural town which doesn’t show the real figure of Ethiopian and need modification | We have now updated the topic in line with your suggestion, indicating the study area. |
| Abstract:   - The 95% Confidence intervals are very wide (ex. Number of children and age), meaning the results are less precise or indication of inadequate sample size - Recommendations are not in line with the result and need revision. The researchers recommend policy formulation. Do you have a sufficient and valid evidence to come up with such type of recommendation or convince policy makers? Do you mean other than the existing intervention policy? | We appreciate that our sample size is small. But we tried to present the results scientifically religious manner. Yes, the small sample size might have affected our confidence intervals. However, our recommendations are now updated in line with our results. |
| Background:   - The knowledge gap on the topic of interest among target study subject is not clearly shown. - The investigators describe a lot about prevalence of malnutrition among children under 5 years. For example, background section (please see paragraph one and paragraph 3, prevalence report from EDHS is for under 5 years children). What is known and unknown about malnutrition among the specified age group (6-12years)? - The authors didn’t work of exhaustive literature search on similar studies done so far (There are several studies even from similar region in the nearby area of the current study). You didn’t cite study on similar population in Ethiopia. Why? Therefore, it is not convincing whether this topic need investigation or not - How about the different efforts to minimize risk of malnutrition or improve nutritional study? Example: Periodic deworming, Vitamin A supplementation, etc…?? | Thanks for all these comments.  - we have now updated the whole background and cited updated reference; but the data on school age children is still scarce  - We have tried to assess the major indicators of undernutrition in the study area.  (Introduction; page-4, starting from line-91) |
| Objective of the study is not clearly shown  Methods   - Even if the study objectives are not indicated, the researchers seem to measure the risk factors of malnutrition. In this case (measuring risk factors) there could be comparison groups. However, you didn’t calculate sample size taking the factors into account using two population proportions etc. thus, the study lacks adequate power to compare different groups | This study is a single population cross-sectional study. It involves a single population, and the sample size was calculated based on single population proportion formula, and 95% CI is our assumption to calculate the sample size. |
| - What procedure did you follow to select study subjects? It is stated that, simple random sampling was used for selecting households. But it is not shown whether census was done or not to get sampling frame. It is well known that there is no sampling frame of houses in Ethiopia. | We used households as a sampling frame. We first obtained the list of households from the city administration and then choose households randomly. |
| - In case two children found in a household, you used the youngest. Why? This could be one of the selection biases introduce in this study | If there were more than one school age children, we studied the younger. It was with the assumption that younger children are more vulnerable to under nutrition. |
| - In the following sentence page 5 line 24 you stated: “***However, we moved to the immediate next household for households 25 without school-age children to conduct our interview***”, given the sampling method, simple random, is it realistic? | We appreciate this comment. Our effort was to reduce absence rate and to get sufficient data as much as possible. |
| - Sometimes the study population is mentioned children 6-12 years, other times under five children. Which one is correct? Please see page 6 (variables of the study & data collection tools section) | School age (children 6-12 years) are our target populations, thank you we have now corrected it. |
| - The method and introduction section is mixed up. Example discussion on the conceptual framework presented on page 7 | We were trying to show variable associations that are examined in our study. |
| - Important but unrelated subheadings are put together and misleading. Example, data analysis, Results, and discussion | We have now corrected it to avoid misleading information. |
| - Under data collection tools and procedure subheading on page 6 it is stated that data was collected on personal behaviours, but the investigators didn’t show on which of the behavioural factor’s measurement done in the independent variable section | Thank you corrected to maternal and child characteristics. |
| - On page 11, ‘*Second, estimates of a child’s nutrition were measured using internationally used standard tools and definitions, which did not consider country specific parameters.* What does this mean?*’* Is it not standard? | We appreciate this. It is to mean the tools used to measure undernutrition are the WHO multicentre standardized tools, but not standardized to specific population; for instance, the parameters used Asian communities is different given that their hight rate for normal children are different than the rest countries. |
| - Did you validate the tools especially anthropometric measurements? If yes, how? | We did not validate the tools. But we used the already validated tool and consistent with other researchers that assess nutritional status in the country. |
| - Most of the variables on which measurement done are something that happened at least before 6 year or at most before 12 years. Example: immunization status, ANC follow-up, additional feeding during pregnancy, history of breast feeding, Vit A supplementation, feeding practice, birth size etc …, and diarrhoea in the last one year. In such circumstances, how did you explain role of introduction of information bias (recall bias) in your findings? | We mentioned this in the limitation part of the study. Since we are studying school age children there could be some information bias related to the time (Page-11-line-299). |
| - Some the variables on which measurement done are vague. Example on table two page 17 and 18: ‘**Do the child eat breakfast regularly**’ what does regularly mean? ‘**Frequency of eating per day**’ on which day? ‘**Water shortage for cooking’ when??** | Thank you we have now put their definition accordingly as a table legend. |
| **Result**   - What is the relevance of collecting highly subjective data? Example most of child and maternal data on page 8 and table 2; Is it relevant to measure relationship between factors happened before 6 years with acute situation? | We appreciate your questions here; but our intension was to show the effects maternal and child characteristics on the level of malnutrition and to show gaps if there, although it has its own limitations of information bias. |
| - Did the researcher check for model fitness and model assumptions? If yes, for which? What are the variable selection criteria for fitting the model? | Model fitness assumption, non-significant Hosmer Lemeshow goodness fit test were checked for variables considered in the multivariable analysis.  (Page-7, line-197-200) |
| - Did you check for multicollinearity? Example, the older the child is the higher probability of work engagement. Furthermore, older age is related with higher level of education. Thus, there could be serious issue in variable selection and model fitting? | Yes, we have checked the multicollinearity and indicated the results in the data analysis section.  (Page-7, line-197-200) |
| - The 95% Cis are very wide. What does this mean? | Thank you for this. We appreciate that this could be due to small sample size of our study. |
| - AOR result is not shown for example in table 3 for some of the variables, why? Whatever the result should be shown | We presented AOR for significant variables in the multivariable analysis section, this to increase the visibility of significant variable for readers**.** |
| **Discussions**   - The investigators compared the result with totally incomparable population and the justification are also not convincing. One simple example: you compared the prevalence of malnutrition for age 6-12 years from the current study with children aged under 5 years in other part of Ethiopia in reference 13 (Discussion section paragraph one). Is it the correct way of discussing your finding? | We appreciate your suggestion here, we have now updated it; although evidence on school age children is still scare in the country. Despite this we have tried to update the comparator literature as much as we can. |
| - Why don’t you discuss in relation to similar setting and population? There are several studies from Ethiopia including the surrounding towns of the current study on similar population. - Did others from similar population support or contradict the risk factors you reported in this study? It is not clear. Very few literatures reviewed, outdated literatures, incomparable results used | We have updated to compare the results in line with currents literature; despite information about school age children are still limited. |
| - Important limitations of the study are not discussed. Example, information bias (anthropometry tools and asking something that happened before 6-12 years)???, Lack of power?? Problems related to model selection and fitting, | Thank you for this suggestion we have now updated the limitation sections to include these points.  (Page-11, line-299). |
| - Recommendations forwarded didn’t take into account the major limitations of the study | We have revised the recommendations based on the limitations to suggest future research. (Page-11, line-324-328). |
| **I don’t understand why the following is not done?**  **‘‘Availability of data and material:** The data of this study cannot be shared publicly due to the presence of sensitive (confidential) participants' information’’, Why?? | This statement is in line with the journal authors guideline. We have the data set at our hand but to share to journal it should fist undergo deidentification to maintain confidentiality and anonymity. We have discussed this with the journals and wrote it in line with their suggestion. |
