# Supplementary material for: Factors driving underweight, wasting, and stunting among urban school aged children: Evidence from Merawi town, Northwest Ethiopia
Source: PLOS Glob Public Health. 2023 Jan 24;3(1):e0000586. doi: 10.1371/journal.pgph.0000586 (PMC10021509; doi:10.1371/journal.pgph.0000586)
Supplement: Attachment — Submitted filename: Responses to reviewers .docx [file pgph.0000586.s003.docx]

**Factors driving underweight, wasting, and stunting among urban school aged children: Evidence from Merawi town, Northwest Ethiopia**

| **Reviewer’s comment** | **Response from Authors** |
| --- | --- |
| The revision done by the authors helped improve the quality of the manuscript. However, the authors failed to provide a scientific reason for not adjusting for the 3 kebeles in their analysis, an issue I raised earlier. Their response that “However, the city has only three kebeles (small administrative units) only used for administrative purposes and the population among the kebeles are homogeneous, there is no difference among them” cannot be supported with the available data in the manuscript, and this must be proven scientifically and presented in the manuscript. Per their response, the authors are saying that the nutritional outcomes of children from the 3 different Kebeles is the same BUT they failed to provide results to support this claim in the manuscript. They should include the kebele as a predictor/covariate in their model. | Thank you for your useful comments. This study was carried out based on data collected from Merawi town, which is a relatively small city having three small administrative units called Kebeles (Local language). We selected participants randomly from the three Kebeles during the conduct of this study. The three Kebeles' populations are homogeneous; thus, we did not sort out and analyse the data based on separated kebeles. Because our sampling frame involved school ages children in the town, participants were randomly selected from all kebeles, not separately from each kebele. |
| In the Abstract and Discussion sections, the authors stated that “prevalence of being underweight, wasting and stunting was found to be 5.7%, 9.8%, 10.4%, respectively”. However, in the ‘Author summary’ section, the authors again stated that “The magnitude of underweight, wasting, stunting was 5.8 %, 10.8%, and 11.6%, respectively” which are completely different. How were the magnitude and the prevalence estimated for which reason the authors were reporting different values? This should be clarified or corrected. | Thank you very much for identifying this typo error. We have now corrected the figures as well as the abstract and results. |
| Also, there are still some typo errors that the authors should correct. For example, ‘under weighs’ in line 235 at page 9. Also, there should be dot (.) before ‘Gender’ in line 235 at page 9. Similar typo error in line 206 at page 11 for ‘motheris’. | Thank you for identifying the typos. We have now addressed the typological errors. |
